# Poly(Ethylene Glycol)-Cholesterol Inhibits L-Type Ca^2+^ Channel Currents and Augments Voltage-Dependent Inactivation in A7r5 Cells

**DOI:** 10.1371/journal.pone.0107049

**Published:** 2014-09-08

**Authors:** Rikuo Ochi, Sukrutha Chettimada, Sachin A. Gupte

**Affiliations:** Department of Biochemistry and Molecular Biology, University of South Alabama, Mobile, Alabama, United States of America; University of Hull, United Kingdom

## Abstract

Cholesterol distributes at a high density in the membrane lipid raft and modulates ion channel currents. Poly(ethylene glycol) cholesteryl ether (PEG-cholesterol) is a nonionic amphipathic lipid consisting of lipophilic cholesterol and covalently bound hydrophilic PEG. PEG-cholesterol is used to formulate lipoplexes to transfect cultured cells, and liposomes for encapsulated drug delivery. PEG-cholesterol is dissolved in the external leaflet of the lipid bilayer, and expands it to flatten the caveolae and widen the gap between the two leaflets. We studied the effect of PEG-cholesterol on whole cell L-type Ca^2+^ channel currents (*I*_Ca,L_) recorded from cultured A7r5 arterial smooth muscle cells. The pretreatment of cells with PEG-cholesterol decreased the density of *I*_Ca,L_ and augmented the voltage-dependent inactivation with acceleration of time course of inactivation and negative shift of steady-state inactivation curve. Methyl-β-cyclodextrin (MβCD) is a cholesterol-binding oligosaccharide. The enrichment of cholesterol by the MβCD:cholesterol complex (cholesterol (MβCD)) caused inhibition of *I*_Ca,L_ but did not augment voltage-dependent inactivation. Incubation with MβCD increased *I*_Ca,L_, slowed the time course of inactivation and shifted the inactivation curve to a positive direction. Additional pretreatment by a high concentration of MβCD of the cells initially pretreated with PEG-cholesterol, increased *I*_Ca,L_ to a greater level than the control, and removed the augmented voltage-dependent inactivation. Due to the enhancement of the voltage-dependent inactivation, PEG-cholesterol inhibited window *I*_Ca,L_ more strongly as compared with cholesterol (MβCD). Poly(ethylene glycol) conferred to cholesterol the efficacy to induce sustained augmentation of voltage-dependent inactivation of *I*_Ca,L_.

## Introduction

Cholesterol, a rigid lipid, embedded in the hydrophobic core of the lipid bilayer with its polar single hydroxyl group on the surface of the membrane, stabilizes the structure of the bilayer [1], [2]. It flip-flops between the external and internal leaflets of the bilayer to establish equilibrated distribution [3], [4]. Cholesterol and sphingomyelin accumulate on lipid rafts with channels and signaling proteins to construct platforms of cellular signaling [5]. Enrichment and depletion of cholesterol utilizing methyl-β-cyclodextrin (MβCD), a cholesterol-binding oligosaccharide [6] have provided evidence to establish that cholesterol is indispensable in the regulation of ion channel function [7]. It regulates the channel activity in lipid media by controlling the physical properties of the bilayer and at the lipid-protein interface by direct interaction with the channel protein [8].

Poly(ethylene glycol) cholesteryl ether (PEG-cholesterol), is a nonionic amphiphile consisting of hydrophobic cholesterol and covalently bound hydrophilic PEG [9]. PEG-cholesterol is water-soluble and is used to formulate lipoplexes to transfect cultured cells [10], and liposomes for encapsulated drug delivery [11]. Since PEG moiety decelerates flip-flop, PEG-cholesterol is accumulated in the outer leaflet of the bilayer in the human skin fibroblast [12], and flattens the caveolae in the K562 human leukemic cell line [13]. The accumulation of PEG-cholesterol produces bumpy protrusions of the external leaflet of the bilayer in human erythrocytes [13], [14]. PEG-cholesterol inhibits raft-dependent endocytosis in HT-1080 human fibrosarcoma cells [9], fibroblasts [12] and leucocytes [13]. The effect of PEG-cholesterol on the function of ion channels has not yet been reported to the best of our knowledge.

L-type Ca^2+^ channel currents (*I*_Ca,L_) through opened Ca_V_1.2 channels supply Ca^2+^ into the muscle to initiate contraction of arterial smooth muscles (ASMCs). In the ASMCs, *I*_Ca,L_ is not generated by action potentials but by moderate sustained depolarization induced by neurotransmitters, hormones, autacoids and mechanical stress [15]. The *I*_Ca,L_ during the sustained depolarization is called window current (*I*_WD_), which is determined by the number of Ca_V_1.2 channels and their voltage-dependent activation and inactivation (VDI) [16], [17]. The enrichment of cholesterol of swine coronary ASMCs by *in vitro* manipulation with cholesterol-saturated MβCD (cholesterol (MβCD)) induces inhibition of *I*_Ca,L_ [18]. Voltage-gated Ca^2+^ channel currents of murine pancreatic β-cells are also inhibited by the enrichment with cholesterol (MβCD) [19]. However, how VDI of Ca_V_1.2 channels are modulated by the cholesterol enrichment has been little clarified.

We showed the effects of PEG-cholesterol on *I*_Ca,L_ of cultured ASMCs, using A7r5 cell-lines derived from embryonic rat thoracic aorta [20]. Loading of PEG-cholesterol induced a long-lived inhibition of *I*_Ca,L,_ similar to the enrichment of cholesterol by cholesterol (MβCD). However, although the cholesterol enrichment did not augment VDI, PEG-cholesterol did augment VDI, and induced an extensive inhibition of *I*_WD_. PEG-moiety replacing hydroxyl group of cholesterol is responsible for the augmentation of VDI. The characteristic effect of PEG-cholesterol on VDI was explained by hypothesizing its localization in the external leaflet of the bilayer.

## Materials and Methods

### Cultured A7r5 cells and pretreatment by the lipids

A7r5 smooth muscle cells were purchased from the American Type Culture Collection (Manassas, VA, USA) and maintained under 5% CO_2_ at 37°C in Dulbecco's modified Eagle's medium with l-glutamine and 4,500 mg/L glucose containing 10% fetal bovine serum (Invitrogen, Carlsbad, CA, USA). They were sub-cultured every 3 or 4 days with trypsin (0.25%) and EDTA (0.02%). A portion of the detached cells were transferred to normal Tyrode's (NT) solution and kept at 4°C in micro-tubes. For patch clamp, the cells in the micro-tube were pretreated at room temperature (21°C) with NT solution as a control, or various concentrations of PEG-cholesterol, cholesterol (MβCD), MβCD or 10 mM PEG dissolved in NT solution for 90–300 min. In the experiment to examine the reversibility of the effects of PEG-cholesterol, cells pretreated with 1 mM PEG-cholesterol for 90 min were further treated with 10 or 30 mM MβCD in the presence of 0.9 or 0.7 mM PEG-cholesterol for longer than 90 min.

### Solutions and drugs

Normal Tyrode's solution contained (in mM): NaCl, 135; KCl, 5.4; CaCl_2_, 1.8; MgCl_2_, 1; Hepes, 5; and glucose, 5.5; and pH was adjusted to 7.4 with NaOH. The Ba^2+^ solution contained (in mM): NaCl, 108; TEACl, 20; CsCl, 5.4; BaCl_2_, 10; MgCl_2_, 1; Hepes, 5; and glucose 5.5; and pH was adjusted to 7.4 with NaOH. 30 µM CdCl_2_ was added to the Ba^2+^ solution or the Ba^2+^ was replaced with 9 mM Mg^2+^ to obtain the background current for subtraction. The pipette solution contained (in mM): Cs-aspartate, 115; TEACl, 20; MgCl_2_, 1; BAPTA, 5; Mg.ATP, 3; GTP, 0.2; and Hepes, 10; and pH was adjusted to pH 7.2 by CsOH. PEG-cholesterol (cholesterol-PEG600), PEG (PEG600), cholesterol (MβCD) and all salts and other drugs were from Sigma-Aldrich (St. Louis, MO, USA).

### Patch-clamp

The A7r5 cells in the micro-tube were dispersed on cover-glass in a chamber mounted on an inverted microscope (IX72, Olympus, Tokyo, Japan). After the attachment of cells to the cover-glass, the chamber was super-fused, first with NT, then with 10 mM Ba^2+^ solution for total ∼40 min (range 12–180 min) before recording the first *I*_Ca,L_. Patch pipettes were pulled from hard glass capillary tubing containing a glass filament using a micropipette puller (P-97 Sutter Instrument Co., Novato, CA, USA) coated with silicone elastomer (Sylgard 184, Dow Corning Co., Midland, MI, USA), and fire-polished using a microforge (MF-830, Narishige, Tokyo, Japan). The pipette resistance was ∼10 MΩ when filled with pipette solution. The voltage-clamp amplifier (Axopatch 200B, Molecular Devices, Sunnyvale, CA, USA) was driven by Clampex 10 software via a digital interface (Digidata 1400, Molecular Devices). Currents were filtered at 2 kHz using the amplifier's low-pass 8-pole Bessel filter, and digitized at 10 kHz before being stored on the computer hard drive for later analysis. Membrane capacitance was obtained by applying a negative-going ramp step, and also by using the built-in program in Clampex. The averaged membrane capacitance was 66 pF. Holding potential (HP) was −80 mV. The *I*_Ca,L_ for the I/V relationship was obtained by applying 500 ms depolarization steps in 10 mV increments at 0.2 Hz from −40 to 50 mV preceded by a 50 or 70 ms pre-pulse to −40 mV to inactivate T-type Ca^2+^ channel currents that exhibited small and variable amplitude. Quasi steady-state inactivation curves (f_∞_/V) were obtained using a gapped double-pulse protocol at 0.1 Hz. A 2 s conditioning pulse to potentials between −100 and 30 mV from a HP of −80 mV was followed by a 50 or 70 ms step to −40 mV, and then a 500 ms test pulse to 0 mV. The *I*_Ca,L_ was quantified after subtracting the background current.

### Data analysis

The *I*_Ca,L_ was analyzed after low-pass filtering with a cut-off frequency of 1 kHz by Clampfit 10 software (Molecular device). Statistical analysis was conducted by GraphPad Prism (V.6, GraphPad Software, San Diego, CA, USA). Igor Pro (V.6, Wavemetrics, Portland, OR, USA) was used for curve fitting and illustration. The I/V relationship of *I*_Ca,L_ was fitted with the following equation adapted from the Boltzmann equation: *I*_Ca,L_  =  G_max_(V-E_rev_)/[1+exp((V_0.5_-V)/k)], where V is the membrane potential, G_max_ is the maximal conductance, E_rev_ is the reversal potential, V_0.5_ is the half activation potential, and k is the slope factor. The f_∞_ (availability)/V relationship was fitted with the following Boltzmann equation: f_∞_ =  c_0_+(c_1_–c_0_)/[1+exp(-(V-V_0.5_)/k)], where: c_0_ is a voltage-independent constant; c_1_–c_0_ is the maximal availability of the voltage-dependent component; V is the membrane potential of pre-pulse; and V_0.5_ is the voltage for 50% inactivation of the voltage-dependent component. Statistical results are presented as mean ± standard error of the mean (S.E.M) in the Figures and mean ± standard deviation (SD.) in the Tables.

## Results

### Effect of PEG-cholesterol, cholesterol (MβCD) and PEG on the I/V relationship and time course of decay of *I*_Ca,L_

**Figure 1:**
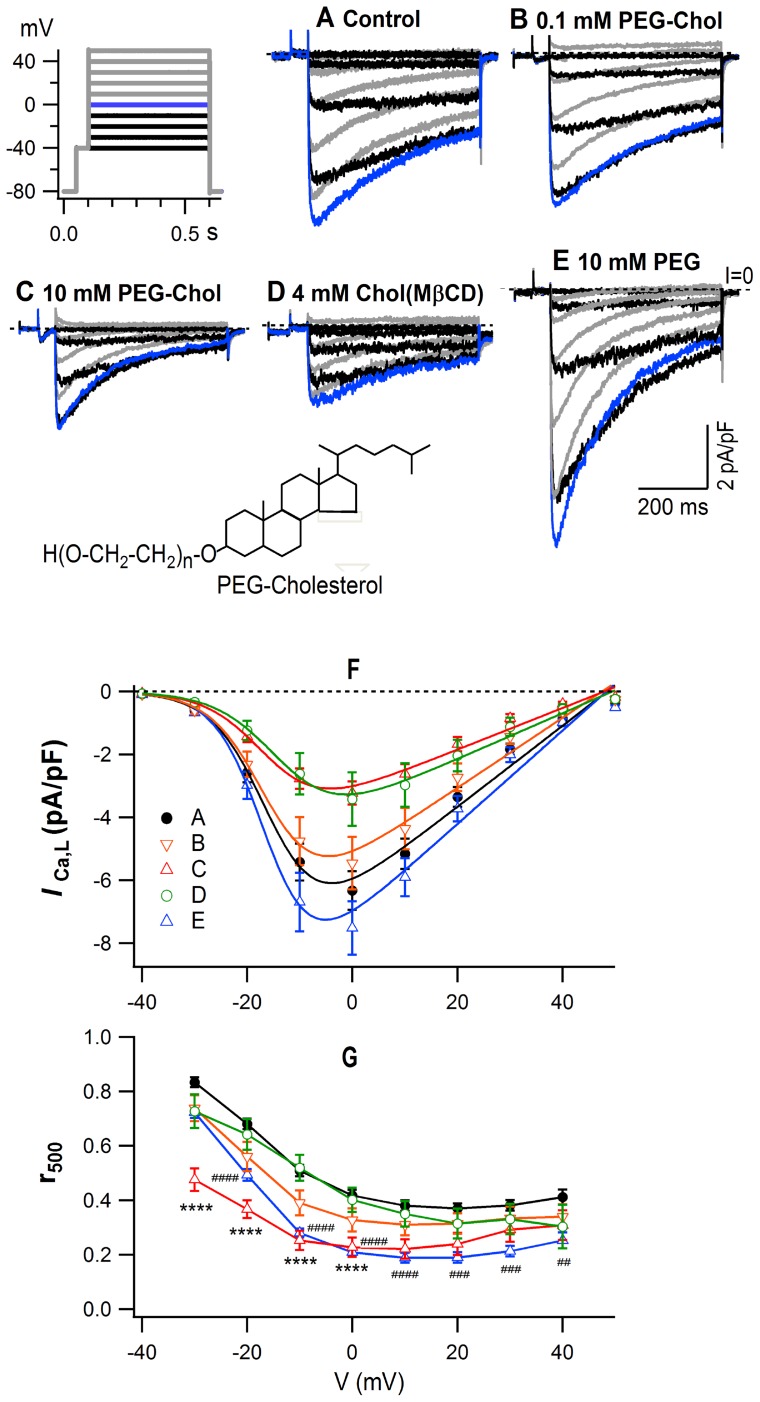
Modulation of *I*_Ca,L_ by pretreatment with PEG-cholesterol, cholesterol (MβCD) and PEG. (**A–E**), typical superimposed current traces. Black traces, between −40 and −10 mV; blue trace, 0 mV; grey traces, >0 mV. Voltage protocol is given in the inset. (**F**), the I/V relationship of the peak *I*_Ca,L_ density (mean ± S.E.M.). Curves were obtained by fitting to the Boltzmann equation (see Methods). n and fitting parameters are given in Table 1. (**G**), the r_500_, the ratio of the amplitude of terminal *I*_Ca,L_ to that of peak *I*_Ca,L_. Statistical comparison was performed using 2way ANOVA followed by Dunnett's test; ##, *p*<0.01, ###, *p*<0.001, ####, ****, *p*<0.0001. Inset, simplified chemical structure of PEG-cholesterol. n, an average number of repeat, was 13.6.

Figure 1 illustrates the voltage-dependent changes of *I*_Ca,L_ from the control cells and cells pretreated with PEG-cholesterol, cholesterol (MβCD) or PEG. Typical current traces (Figures 1A–E) show that *I*_Ca,L_ increased with an increase of depolarization, reached a maximum amplitude near 0 mV, and decreased with further increase of the depolarization to reach a reversal potential near 50 mV. PEG-cholesterol inhibited *I*_Ca,L_ slightly at 0.1 mM, and induced a marked inhibition at 10 mM associated with an accelerated time course of current decay during the depolarization. 4 mM of cholesterol (MβCD), induced an intensive inhibition of *I*_Ca,L_ without accelerating the time course of current decay. PEG600 (10 mM) slightly increased the peak amplitude with a clear acceleration of the time course of current decay.

The amplitude of *I*_Ca,L_ was estimated after subtracting the background current and plotted against voltage (V). Figure 1F shows the I/V relationship of the peak *I*_Ca,L_ density. The maximum density was obtained at 0 mV. It was 6.3±0.6 pA/pF (mean ± S.E.M., n = 37) in the control, decreased with the pretreatment with PEG-cholesterol and cholesterol (MβCD), and increased with 10 mM PEG. The I/V relationship was fitted by the Boltzmann equation. The reversal potential of *I*_Ca,L_ (E_rev_) was 48.4±2.2 mV in the control, and was little affected by the pretreatments (Table 1). The amplitude of *I*_Ca,L_ in the curve fitting was maximal between 0 and −10 mV, except for that from 4 mM cholesterol (MβCD)-pretreated cells (Figure 1F). G_max_, the maximal conductance of *I*_Ca,L_, was 128.9±11.3 pS/pF in the control, decreased by 14% with 0.1 mM PEG-cholesterol, 49% with 10 mM PEG-cholesterol, 43% with 4 mM cholesterol (MβCD), and increased by 15% with 10 mM PEG (Table 1). The V_0.5_ for activation was −15.3±1.3 mV in the control, and shifted to a depolarizing direction by 2.2 mV with 4 mM cholesterol (MβCD) (Table 1). The rate of the current decay during the pulse was quantified by r_500_, the ratio of *I*_Ca,L_ at 500 ms of the pulse to the peak amplitude. The r_500_ was little affected with 4 mM cholesterol (MβCD), but decreased with PEG-cholesterol in a concentration-dependent manner to ∼50% with 10 mM PEG-cholesterol, and also with 10 mM PEG600 with a statistical significance when compared with the control (Figure 1G).

**Table 1:** The effect of PEG-cholesterol, PEG, cholesterol (MβCD) and MβCD on parameters of the I/V relationship.

	n	G_max_(pS/pF)	V_0.5_ (mV)	k (mV)	E_rev_(mV)
Control	37	128.9±11.3	−15.3±1.3	5.1±0.9	48.4±2.2
0.1 mM PEG-cholesterol	9	111.5±0.8	−15.9±1.4	5.1±1.0	47.5±2.4
1 mM PEG-cholesterol	15	72.8±7.1*	−16.8±1.4	5.0±1.0	47.7±2.5
10 mM PEG-cholesterol	9	66.3±6.7*	−15.8±1.6	5.5±1.1	47.9±2.5
10 mM PEG600	14	148.6±14.4*	−16.0±1.4	4.6±1.0	48.3±2.6
4 mM cholesterol (MβCD)	9	73.0±6.4*	−13.1±1.4*	5.8±0.9	49.4±2.1
1 mM MβCD	10	172.0±15.2*	−17.6±1.3*	5.2±1.0	48.2±2.3
10 mM MβCD	16	210.2±18.5*	−17.0±1.3	4.6±0.9	48.1±2.8
30 mM MβCD	17	178.2±16.0*	−15.6±1.3	5.2±0.9	49.1±2.3
1 mM PC,10 mM MβCD	4	70.4±7.9*	−16.0±1.7	5.4±1.2	47.7±2.3
1 mM PC,30 mM MβCD	13	208.6±20.5*	−20.1±1.5*	5.0±1.1	46.1±2.6

Mean values of *I*_Ca,L_ density were plotted against test potential (V) and applied to the Boltzmann equation to obtain parameters (see, Methods). 1 mM PC reads pretreatment by 1 mM PEG-cholesterol. n, number of cells. Mean ± SD values. Statistical comparison was performed using ordinary one-way ANOVA followed by Dunnett's test; *, *p*<0.01.

### Effect of PEG-cholesterol, cholesterol (MβCD) and PEG on steady-state inactivation of *I*_Ca,L_

**Figure 2:**
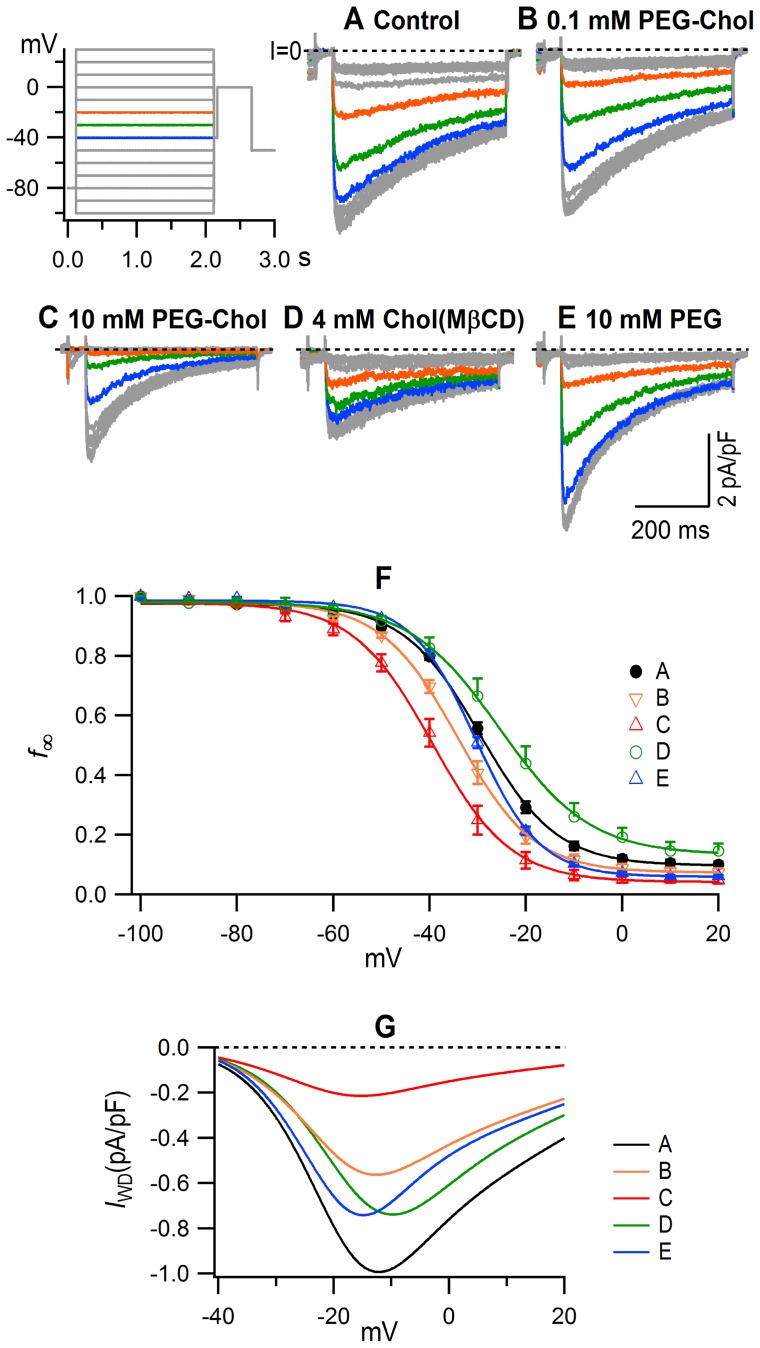
Modulation of voltage-dependent inactivation by pretreatment with PEG-cholesterol, cholesterol (MβCD) and PEG. (**A–E**), typical superimposed current traces. The inset illustrates voltage protocol. The blue, green and orange traces illustrate current and voltage traces for −40, −30 and −20 mV, respectively. (**F**), the f_∞_/V relationship obtained by plotting the ratio (f_∞_) of peak amplitude of *I*_Ca,L_ to its maximal amplitude against conditioning potential (V). Curves were obtained by fitting to the Boltzmann equation. n and fitting parameters are given in Table 2. (**G**), the *I*_WD_/V relationship. The *I*_WD_ was obtained by multiplying values of *I*_Ca,L_ from simulated I/V relationships (Figure 1**F**) and f_∞_ from simulated f_∞_/V relationships (Figure 2**F**). Data were obtained from the same cells in Fig. 1.

Figure 2 illustrates the effects of the pretreatments with PEG-cholesterol and others on quasi-steady-state voltage-dependent inactivation. The *I*_Ca,L_ was gradually inactivated by the increase in depolarization of the pre-pulse. The pre-pulse-dependent inactivation increased steeply between −40 mV and −20 mV (Figures 2 A–E). The peak current amplitude of the *I*_Ca,L_ normalized by the maximal amplitude was plotted against the pre-potential to obtain the quasi-steady-state inactivation relationship (f_∞_/V)(Figure 2F). The relationships were sigmoidal, with a small voltage-independent component that was not inactivated by large depolarization. The f_∞_/V relationships were well-fitted by the Boltzmann equation with the parameters given in Table 2. In the control, the V_0.5_ was −29.1±0.4 mV and the voltage-independent availability (c_0_) was 0.11±0.01. PEG-cholesterol shifted the curve to the left, making the V_0.5_ more negative at −34.2 mV at 0.1 mM, and −39.3 mV at 10 mM, and decreased the c_0_ in a concentration-dependent manner (Table 2). In contrast, 4 mM cholesterol (MβCD) increased the c_0_ and shifted the curve in a depolarizing direction. 10 mM PEG slightly shifted the curve in a hyperpolarizing direction (V_0.5_, −30.3 mV), steepened the slope of the curve, and decreased the c_0_.

**Table 2:** The effect of PEG-cholesterol, PEG, cholesterol (MβCD) and MβCD on parameters of the f_∞_/V relationships.

	n	c_0_	c_1_–c_0_	V_0.5_ (mV)	k (mV)
Control	37	0.11±0.01	0.87±0.01	−29.1±0.4	7.8±0.4
0.1 mM PEG-cholesterol	9	0.07±0.01*	0.91±0.01*	−34.2±0.4*	7.9±0.4
1 mM PEG-cholesterol	15	0.04±0.01*	0.93±0.01*	−39.3±0.4*	7.7±0.4
10 mM PEG-cholesterol	9	0.04±0.01*	0.94±0.01*	−39.5±0.5*	8.2±0.4
10 mM PEG600	14	0.06±0.01*	0.93±0.01*	−30.5±0.3*	6.7±0.2*
4 mM cholesterol (MβCD)	9	0.13±0.01*	0.85±0.01*	−25.5±0.4*	9.4±0.3*
1 mM MβCD	10	0.08±0.01	0.89±0.01*	−29.9±0.4*	8.2±0.4
10 mM MβCD	16	0.12±0.01	0.86±0.01	−26.6±0.4*	7.4±0.4
30 mM MβCD	17	0.17±0.02*	0.82±0.01*	−24.3±0.5*	9.3±0.4*
1 mM PC,10 mM MβCD	4	0.04±0.01*	0.94±0.01*	−45.3±0.5*	7.3±0.4*
1 mM PC,30 mM MβCD	13	0.10±0.01	0.86±0.01	−34.6±0.6*	9.1±0.5*

Amplitude of *I*_Ca,L_ elicited by a constant test pulses after conditioning pulses were normalized by the maximal amplitude as f_∞_and the mean values of f_∞_ were plotted against conditioning potential (V) and were fitted to the Boltzmann equation (see Methods). 1 mM PC reads 1 mM PEG-cholesterol. n, number of cells. Mean ± SD values. Statistical comparison was performed using ordinary one-way ANOVA followed by Dunnett's test; *, *p*<0.01.

**Figure 3:**
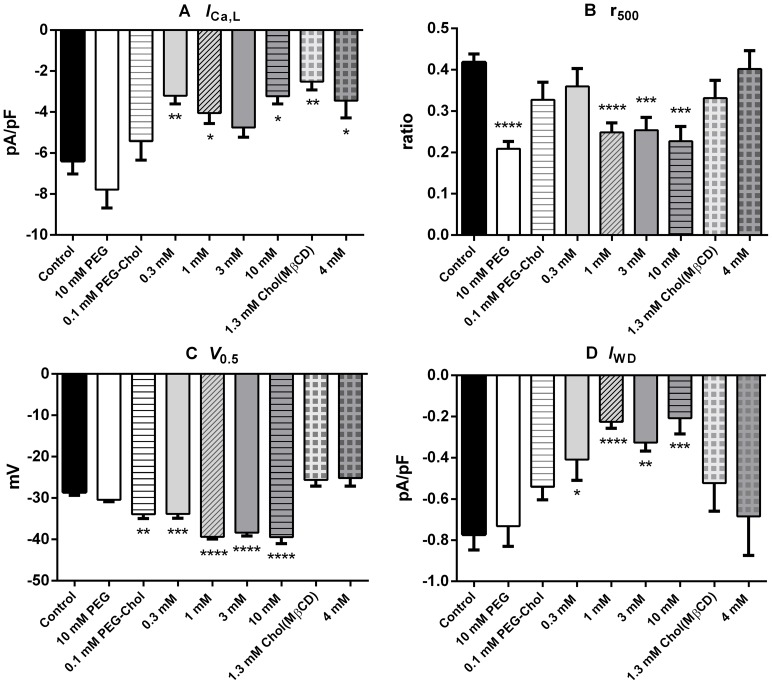
Summarized effects of PEG-cholesterol, PEG and cholesterol (MβCD) on *I*_Ca,L_, r_500_, V_0.5_ of f_∞_/V and *I*_WD_. (**A**), Maximal *I*_Ca,L_ density; (**B**), r_500_ obtained at 0 mV (**C**), the V_0.5_ of f_∞_/V relationship as averaged value of that obtained in each experiment. (**D**), averaged value of maximal density of the *I*_WD_ obtained in each experiment by multiplying *I*_Ca,L_ density and f_∞_ value. The numerical values represent mean ± S.E.M. n: control, 37; 10 mM-PEG, 14; PEG-cholesterol; 0.1 mM, 9, 0.3 mM, 14, 1 mM, 15, 3 mM, 12 and 10 mM, 9; cholesterol (MβCD): n = 9 for both 1.3 and 4 mM. Statistical comparison was performed using ordinary one-way ANOVA followed by Dunnett's test; *, *p*<0.05, **, *p*<0.01, ***, *p*<0.001, ****, *p*<0.0001.

From the I/V (Figure 1) and the f_∞_/V relationships, the *I*_WD_ was calculated as the product of the simulated curves (I·f_∞_/V) at a voltage range between −40 and 20 mV (Figure 2G). In the control, the *I*_WD_ density was maximal at a slightly negative voltage of −10 mV, as large as ∼80% of the maximal density at −20 mV, and ∼30% at −30 mV. PEG-cholesterol strongly inhibited *I*_WD_ in a concentration-dependent manner. It inhibited the *I*_WD_ more extensively compared with its inhibition of *I*_Ca,L_, i.e. the ratio of maximal *I*_Ca,L_ to maximal *I*_WD_ was 0.12 in the control and 0.09 and 0.06 in 0.1 and 10 mM PEG-cholesterol pretreated cells respectively. 4 mM cholesterol (MßCD) induced relatively small inhibition of the *I*_WD_ with the maximal *I*_WD_ to maximal *I*_Ca,L_ ratio of 0.23, as it shifted the f_∞_/V relationship to the right and increased the c_0_. Figure 3 summarizes the changes in the values of *I*_Ca,L_, r_500_, V_0.5_ and *I*_WD_ obtained with several concentrations of PEG-cholesterol, PEG and cholesterol (MβCD). *I*_Ca,L_ given by the maximal current density was inhibited by PEG-cholesterol in a concentration-dependent manner (Figure 3A). It was slightly increased by 10 mM PEG, and was inhibited by 1.3 and 4 mM cholesterol (MßCD) almost to the same extent. Correspondingly, the G_max_ that was almost proportional to the maximal current density of *I*_Ca,L_ was slightly increased by 10 mM PEG, decreased by PEG-cholesterol in a concentration-dependent manner, and was decreased by cholesterol (MβCD (partly shown in Table 1). The r_500_ estimated at 0 mV decreased with PEG and 1, 3 and 10 mM PEG-cholesterol, with statistical significance, but was not significantly affected by 1.3 and 4 mM cholesterol (MβCD) (Figure 3B). The V_0.5_ of f_∞_/V relationship was shifted to more negative potentials in a concentration-dependent manner by PEG-cholesterol but was slightly shifted in a depolarizing direction by 1.3 and 4 mM cholesterol (MβCD), and was slightly shifted in a negative direction by 10 mM PEG (Figure 3C, Table 2). The *I*_WD_ was concentration-dependently inhibited by PEG-cholesterol more strongly when compared with the inhibition of *I*_Ca,L_, while it was not affected with statistical significance by 1.3 and 4 mM cholesterol (MβCD) and 10 mM PEG pretreatment (Figure 3D).

### Effects of MβCD on *I*_Ca,L_ modulated by PEG-cholesterol

**Figure 4:**
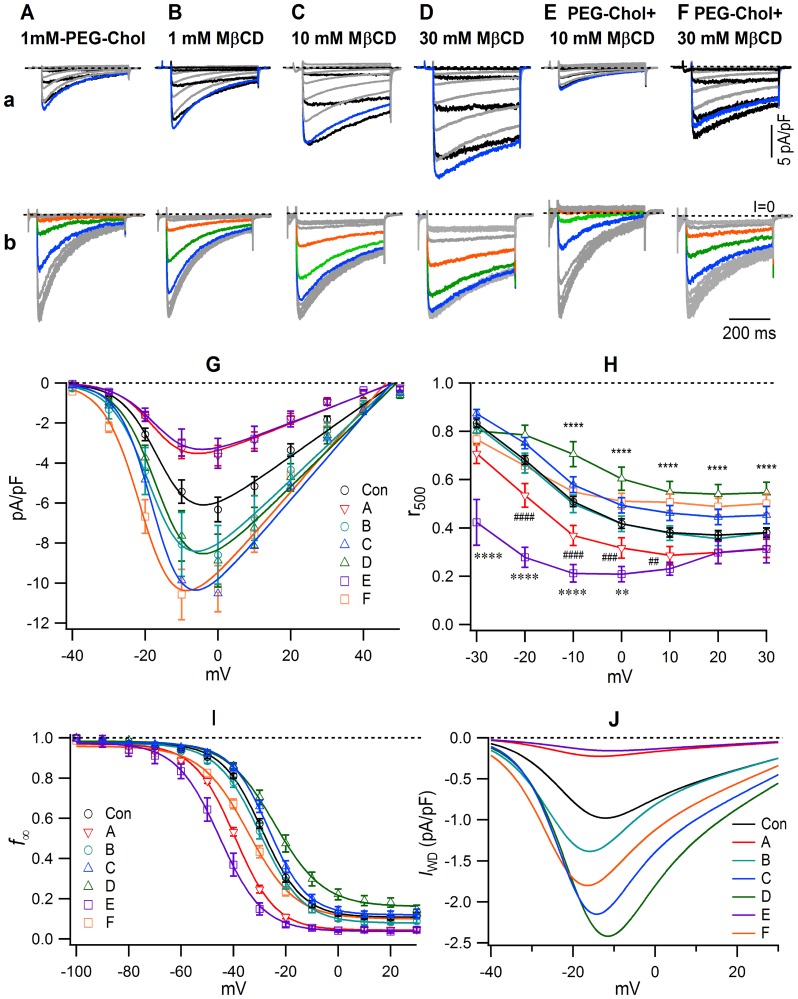
The effect of MβCD on control *I*_Ca,L_ and on *I*_Ca,L_ modulated by PEG-cholesterol. (**a**), typical current traces for I/V relationships; (**b**), typical non-calibrated current traces for f*_∞_*/V relationships; voltage protocols and colors with difference in voltage are depicted in Figure 1 and 2. Pretreatment by (**A**), 1 mM PEG-cholesterol; (**B**), 1 mM MβCD; (**C**), 10 mM MβCD; (**D**), 30 mM MβCD; (**E**), (**F**), initially by 1 mM PEG-cholesterol, and then by 10 (**E**) and 30 mM MβCD (**F**). (**G**), the I/V relationship of the peak current density. (**H**), the r_500_/V relationship. Statistical comparison was performed using 2way ANOVA followed by Dunnett's test; **, **, *p*<0.01, ***, *p*<0.001, ****, ****, ####, *p*<0.0001. (**I**), the f_∞_/V relationship. The I/V and f_∞_/V relationships were fitted to the Boltzmann equations and the constants obtained from the fitting are given in Table 1 and Table 2. (**J**), the I_WD_/V relationship. The *I*_WD_ was obtained by multiplying simulated *I*_Ca,L_/V and f_∞_/V relationships.

Pretreatment with MßCD, a scavenger of cholesterol, increased *I*_Ca,L_ (Figures 4B–D, G, Table 1) and slowed the time of inactivation (Figure 4H) in a concentration-dependent manner. The pretreatment with MβCD also shifted the f_∞_/V relationship in the depolarizing direction and increased the c_0_ in a concentration-dependent manner (Figure 4I, Table 2), i.e., in the 10 and 30 mM MβCD pretreated cells, *I*_Ca,L_ was only slightly affected by the conditioning pulse at −40 mV, and large currents appeared even after the conditioning by a −20 mV pre-pulse (Figure 4b). PEG-cholesterol (1 mM) induced ∼50% inhibition of *I*_Ca,L_ amplitude (Figure 4G), accelerated the current decay (Figure 4A, H), shifted the f_∞_/V relationship to the left by 10 mV, and decreased the c_0_ (Figure 4I, Table 2). We examined the effects of 10 and 30 mM MβCD on PEG-cholesterol-induced modulation of *I*_Ca,L_. Additional pretreatment with 10 mM MβCD of the 1 mM PEG-cholesterol pretreated cells did not reverse the inhibition of the current density (Figure 4Ea, G), the acceleration of the time course of decay (Figure 4H), or the negative shift of the V_0.5_ (Figure 4I). However, the pretreatment with 30 mM MβCD of the PEG-cholesterol pretreated cells produced a large increase of *I*_Ca,L_ (Figure 4Fa, G) associated with a shift of the I/V relationship to the left, with a shift of the V_0.5_ from the control's −15.3 to −20.1 mV (Table 1). Furthermore, 30 mM MβCD reversed PEG-cholesterol-induced acceleration of the time course of inactivation (Figure 4F, H) and partially reversed the PEG-cholesterol-induced leftward shift of the V_0.5_ (Figure 4I) associated with a recovery of the c_0_ to the control value (Table 2). Reflecting the changes of the I/V and f_∞_/V relationships, the *I*_WD_ was largely inhibited by 1 mM PEG-cholesterol, markedly increased by MβCD in a concentration-dependent manner, and the PEG-cholesterol-induced inhibition was not recovered by the additional pretreatment by 10 mM MβCD, but was increased to more than the control by that of 30 mM MβCD (Figure 4J).

## Discussion

Amphipathic PEG-cholesterol inhibited *I*_Ca,L_, augmented VDI and strongly inhibited the *I*_WD_. PEG-moiety covalently bound to cholesterol was responsible for the augmentation of VDI, since cholesterol enrichment by cholesterol (MβCD) did not augment VDI. The effects of PEG-cholesterol on *I*_Ca,L_ were long-lived, and were apparently reversed by MβCD.

### Inhibition of *I*_Ca,L_ by cholesterol and PEG-cholesterol

Enrichment of cholesterol using cholesterol (MβCD) induces strong inhibition of *I*_Ca,L_ in guinea pig gallbladder smooth muscle cells [21]. *I*_Ca,L_ of cholesterol-enriched coronary ASMCs from swine with hypercholesterolemia, and those of cholesterol (MβCD)-treated ASMCs from normal swine, are ∼50% of the control in the current density without change in the voltage-dependence of activation, and the inhibition is reversible by MβCD [18]. In the present study, the enrichment of cholesterol by cholesterol (MβCD) and the loading of PEG-cholesterol decreased the maximal amplitude of *I*_Ca,L_ and G_max_ by ∼50%. Contrastingly, the removal of cholesterol by MβCD from the membrane clearly increased *I*_Ca,L_. Voltage-gated Ca^2+^ channel curren is augmented by MβCD also in chick cochlear hair cells [22]. The cholesterol (MβCD)-induced inhibition and MβCD-induced augmentation suggest that endogenous cholesterol constitutively inhibits *I*_Ca,L_ as proposed by the MβCD-induced augmentation of Ca^2+^-permeable transient receptor potential melastatin (TRPM)3 activity [23]. The mechanisms of the cholesterol enrichment- and PEG-cholesterol- induced inhibition of *I*_Ca,L_ are elusive. The thickness of the lipid bilayer of rabbit ASMC increases with dietary cholesterol enrichment [24]. Single channel conductance of BK_Ca_ (*hSlo* α-subunit) channels in planar lipid bilayers decreases with the increase of bilayer thickness [25]. PEG-cholesterol loaded on the external leaflet increases the bilayer thickness, as the PEG-tail extends over the surface of the external leaflet and PEG-cholesterol insertion expands the external leaflet to induce a separation of the bilayer [13]. The thickening of the bilayer produced by cholesterol (MβCD) and PEG-cholesterol may decrease the unitary conductance of Ca_V_1.2 channels. The conductance and the open probability of the single Ca_V_1.2 channel currents in the cholesterol (MβCD) and PEG-cholesterol treated cells should be clarified.

The Ca_V_1.2 channel complex that generates *I*_Ca,L_ is consisted of α_1_, β and α_2_δ subunits in ASMCs, cardiac myocytes, neurons and endocrine cells [26]. The α_1_ subunit is the primary subunit with a voltage-dependent gate and ion-selective pore, and the β and the α_2_δ are accessory subunits to modulate the gating of the α_1_ subunit and assist its trafficking to the plasma membrane. The α_2_ subunit is exposed over the external surface and the δ subunit is bound to the α_2_ subunit by a disulfide bond at one end, and is fixed to the outer leaflet at the other end [27], [28]. Pregabalin, an α_2_δ ligand, induces the inhibition of *I*_Ca,L_ in rat cerebral ASMCs [29] and the α_2_δ –1 subunit is essential for the plasma membrane expression of the α1 subunit [28], [29]. The PEG-cholesterol solubilized in the outside of the membrane as well as in the external leaflet may directly interact with the α_2_δ subunit to down-regulate its regulatory roles to maintain *I*_Ca,L_.

PEG-cholesterol-induced inhibition was apparently reversible by 30 mM (but not by 10 mM) MβCD. The necessity of the high concentration may be explained by the difficulty to remove membrane-embedded PEG-cholesterol by MβCD, since the size of PEG-cholesterol is larger than that of cholesterol and PEG is hydrophilic. However, the MβCD –induced reversal could be produced by the removal of cholesterol and its non-specific effects to remove the membrane lipids other than cholesterol [6]. PEG600 applied at a high concentration of 10 mM as negative control of PEG-cholesterol did not induce inhibition of *I*_Ca,L_.

### Modulation of VDI by cholesterol (MβCD) and PEG-cholesterol

The VDI of N-type Ca^2+^ channel currents (*I*_Ca,N_) is not affected by the enrichment of cholesterol by cholesterol (MβCD) in the neuroblastoma-glioma hybrid cells [30]. The time course of inactivation of voltage-gated Ca^2+^ channel currents of murine pancreatic β-cells is not affected by the cholesterol enrichment by cholesterol (MβCD) [19]. However, exposure of IMR32 neuroblastoma cells to a cholesterol-enriched medium with tetrahydrofuran for 20–24 hours shifts the V_0.5_ of the steady-state inactivation-voltage relationship of *I*_Ca,N_ ∼20 mV in a depolarizing direction [2]. In the present study, both the enrichment and the depletion of cholesterol performed using MβCD shifted the V_0.5_ in a depolarizing direction associated with an increase of the c_0_ (Table 2). The increase of the c_0_ was reflected in the slow current decay in the MβCD-pretreated cells (Figure 4). MβCD could scavenge membrane lipids other than cholesterol by non-specific binding to counteract the VDI [6]. Nevertheless, since the enrichment and the depletion of cholesterol using MβCD modulated the density of *I*_Ca,L_ differently, we consider that the cholesterol enrichment does not augment the VDI. The pretreatment by a high concentration of PEG600 unexpectedly accelerated the time of inactivation (Figure 1 and Figure 3), although it only slightly affected the I/V and f_∞_/V relationships. The detailed effects of PEG600 and the underlying mechanism should be studied more in the future.

PEG-cholesterol augmented the VDI of *I*_Ca,L,_ manifested with the decrease of the r_500_, the decrease of the c_0_ and the negative shift of the f_∞_/V relationship (Figure 2, Table 2). The augmentation of the VDI could arise both from conformational changes of the Ca_V_1.2 channel complex and the changes of the expression of subunits of the complex. The hypothetical PEG-cholesterol-induced thickening of the hydrophobic core of the bilayer in the presence of constant hydrophobic length of the *α*_1_ subunit induces a lipid-protein hydrophobic mismatch [31]. The mismatch forces the bending of the bilayer adjacent to the *α*_1_ subunit to re-align the lipid bilayer hydrophobic core to the subunit's hydrophobic exterior. It induces a change of configuration of the *α*_1_ subunit, which could result in the augmentation of the VDI. Co-expression of the α_2_δ subunit with the α_1_ and β subunits augments the VDI in Ca_V_1.2 and Ca_V_2.1 channels extrinsically expressed in HEK293 cells [32]. PEG-cholesterol pretreatment could compromise the facilitatory action of the α_2_δ subunit in the α_1_ subunit trafficking to the membrane [28], [29] to result in a relative abundance of the α_2_δ subunit, and thus the augmentation of the VDI. As another mechanism, PEG-cholesterol or PEG-cholesterol-induced expansion of the external leaflet [12] could physically stimulate the α_2_δ subunit to augment the VDI.

Triton X-100 (TX-100) is an amphiphile possessing poly [oxy-ethylene glycol] chain, analogous to PEG. TX-100 reversibly inhibits *I*_Ca,L_ in non-neuronal cells, including rat mesenteric artery ASMCs, with IC_50_ of low µmoles [33]. Moreover, it rapidly and reversibly augments the VDI of *I*_Ca,N_ with ∼20 mV negative shift of steady-state inactivation curve in IMR32 neuroblastoma cells [2]. TX-100 and other amphiphiles, such as capsaicin, shift the V_0.5_ of the Na channel current in a hyperpolarizing direction in HEK293 cells with an extrinsic expression of Na^+^ channels [34]. However, the observation that water-soluble PEG-cholesterol can be encapsulated in liposomes constructed by phosphatidylcholine indicates that PEG-cholesterol does not have detergent-like activity [13]. The pretreatment with 10 mM PEG shifted the f_∞_/V relationship in a hyperpolarizing direction only slightly (Figure 2, Table 2), and its acute application did not affect the f_∞_/V relationship in a preliminary experiment. Therefore, the covalent coupling with cholesterol was necessary for PEG to induce the inhibition of *I*_Ca,L_ and the augmentation of the VDI.

To summarize the mechanism, solvation of PEG-cholesterol into the outer leaflet of the lipid bilayer expands the leaflet and increases thickness of the bilayer. It can induce a hydrophobic mismatch between the Ca_V_1.2α_1_ subunit and the bilayer in A7r5 cells. The mismatch could induce configurational change of the α_1_ subunit. Also, the configuration of the α_2_δ subunits may be changed by direct interaction with PEG-cholesterol or the expansion of the external leaflet. We hypothesize that these changes of Ca_V_1.2 configurations and those of the subunit expression induce the PEG-cholesterol-induced augmentation of the VDI.

In conclusion, PEG-cholesterol inhibited *I*_Ca,L_ and augmented its voltage-dependent inactivation (VDI). The augmentation of the VDI contributed to the PEG-cholesterol-induced strong inhibition of *I*_WD_. Poly(ethylene glycol) conferred to cholesterol the efficacy to induce sustained augmentation of VDI of *I*_Ca,L_.
